# A Review of the Changes Produced by Extrusion Cooking on the Bioactive Compounds from Vegetal Sources

**DOI:** 10.3390/antiox12071453

**Published:** 2023-07-19

**Authors:** Silvia Mironeasa, Ionica Coţovanu, Costel Mironeasa, Mădălina Ungureanu-Iuga

**Affiliations:** 1 Faculty of Food Engineering, “Ştefan cel Mare” University of Suceava, 13 Universitatii Street, 720229 Suceava, Romania; silviam@fia.usv.ro (S.M.); ionica.cotovanu@usm.ro (I.C.); 2 Faculty of Mechanical Engineering, Automotive and Robotics, “Ştefan cel Mare” University of Suceava, 13 Universitatii Street, 720229 Suceava, Romania; costel.mironeasa@usm.ro; 3 Integrated Center for Research, Development and Innovation in Advanced Materials, Nanotechnologies and Distributed Systems for Fabrication and Control (MANSiD), “Ştefan cel Mare” University of Suceava, 13 Universitatii Street, 720229 Suceava, Romania; 4 Mountain Economy Center (CE-MONT), “Costin C. Kiriţescu” National Institute of Economic Researches (INCE), Romanian Academy, 49 Petreni Street, 725700 Vatra Dornei, Romania

**Keywords:** antioxidants, cereals and pseudo-cereals, extruded snacks, fibers, fruits and vegetables, herbs, polyphenols, terpenes, vitamins, minerals

## Abstract

The demand for healthy ready-to-eat foods like snacks is increasing. Physical modification of vegetal food matrices through extrusion generates significant changes in the chemical composition of the final product. There is a great variety of food matrices that can be used in extrusion, most of them being based on cereals, legumes, fruits, vegetables, or seeds. The aim of this review was to summarize the main effects of the extrusion process on the bioactive compounds content, namely phenolics, terpenes, vitamins, minerals, and fibers of vegetal mixes, as well as on their biological activity. The literature reported contradictory results regarding the changes in bioactive compounds after extrusion, mainly due to the differences in the processing conditions, chemical composition, physicochemical properties, and nutritional value of the extruded material and quantification methods. The thermolabile phenolics and vitamins were negatively affected by extrusion, while the fiber content was proved to be enhanced. Further research is needed regarding the interactions between bioactive components during extrusion, as well as a more detailed analysis of the impact of extrusion on the terpenes since there are few papers dealing with this aspect.

## 1. Introduction

Fruits, vegetables, nuts, seeds, and cereals are highly recommended to be included in the human diet since they are nutritious and contain bioactive compounds with many health benefits, including prevention and amelioration of some diseases [1]. Food processing through extrusion cooking determines changes in products’ chemical composition, physical properties, and sensory profile. The content of bioactive compounds from vegetal foods depends on the manufacturing conditions, storage, packaging, and transport decreases of phytonutrients being observed [2]. On the other hand, there are processing techniques such as frying, extrusion, semi-cooking, and steaming that determine the increase in heat-stable bioactive compounds availability [2]. The proximate composition of the raw material influences the extrusion process, with dietary fiber, minerals, and pH modifiers being among the components that contribute significantly despite their small concentrations [3]. Before extrusion cooking, the raw materials are ground to form flour or grits that are usually rich in carbohydrates. High temperature and shear during extrusion generate modifications of the molecular structure of food ingredients biopolymers which result in a great range of snack properties [4]. Among the carbohydrates present in the ingredients used in extrusion, starch plays an essential role in the expansion, while fibers have a small contribution to the expansion improvement, a fact that usually determines their use in limited amounts [5,6].

The most common grains and seeds used for obtaining extruded snacks are corn, wheat, rice, oats, barley, rye, triticale, and sorghum from the cereals category, buckwheat, amaranth, quinoa, chia, from pseudocereals, and soybeans, chickpeas, dry beans, cowpeas, peas, and, lentils from legumes and pulses category, linseed, peanuts, pumpkin seed, sesame seed, and flaxseed from oily seeds category [7]. A mixture between cereals and other vegetal products like legumes, fruits, and vegetables is often used in extrusion due to the enrichment of the protein, dietary fiber, micronutrient, and phytochemical profiles of the final product [8]. The use of industrial ingredients such as “fruit juice, pulp and powder, vegetable powder, dried pieces of fruits and vegetables, and dried ground leafy vegetables” [9] is promising for obtaining novel functional products.

Fruits contain significant amounts of vitamins and pro-vitamins, such as vitamin C and pro-vitamin A, while vegetables are richer in minerals like phosphorus (P), sulfur (S), iron (Fe), and calcium (Ca) [10]. Apart from vitamins and minerals, fruits and vegetables also have an important content of phytochemicals responsible for health-promoting characteristics, including the anticarcinogenic effect [10]. The food industry generates high amounts of vegetal by-products with high nutritional potential that can be used in extrusion cooking to create value-added snacks. These by-products are rich sources of dietary fiber, proteins, and bioactive molecules like phenolic compounds, minerals, and vitamins [11]. Legume seeds are important in human nutrition not only due to their nutritional profile but also due to their content of bioactive compounds such as flavonoids, tocopherols, carotenoids, fatty acids, and anthocyanins [1]. The consumption of legume seeds led to a decrease in low-density lipoprotein (LDL) cholesterol quantity, prevention of heart problems, gastro-intestinal carcinoma, diabetes, cerebrovascular accident, and hypercholesterolemia [1]. Apart from the high amount of soluble and insoluble carbohydrates found in cereals, they also contain vitamins, minerals, phenolic compounds, carotenoids, tocopherols, anthocyanins, and phytosterols that contribute to the beneficial health effects such as immunomodulatory activity, antioxidant properties, antiproliferative and hepatoprotective character [12]. Pulses are sources of proteins, sugars, dietary fibers, vitamins, minerals, oligosaccharides, isoflavones, phospholipids, carotenoids, phytic acid, sterols, saponins, and compounds with antioxidant activity, which have demonstrated implications in prevention and amelioration of diabetes, obesity, cancer, osteoporosis, and cardiovascular system problems [13]. Spices and herbs can be used to enhance the functional value of snacks due to their bioactive compounds like sulfur-containing molecules, tannins, alkaloids, vitamins, flavonoids, and polyphenols that contribute to the antioxidant activity, anti-inflammatory and anticarcinogenic effects [14].

The multitude of interactions among the extrusion processing conditions like temperature, die dimension, screw configuration and speed, feed moisture content and feeding rate, and system parameters such as torque, pressure, and specific mechanical energy are the key factors in affecting the characteristics of the final product [15]. Therefore, it becomes important to understand the variety of changes that the individual components undergo during extrusion, to effectively use raw ingredients or incorporate by-products into the extruded products.

This review aimed to evidence the effects of the extrusion cooking of cereal, legumes, fruits, vegetables, seeds flours, or their composite flours on the bioactive compounds such as phenolics, terpenes, vitamins, minerals, and fibers, and on their biological activity. This information is of great importance for the food industry for further optimizations of novel products, for consumers to know the variety of products that can be found on the market, and for the research community to support the development of new functional food prototypes.

## 2. Overview of the Extrusion Process

Extrusion cooking represents one of the most convenient food processing technologies for food and feed industries. Over the years, extrusion cooking has been applied for the development of various snacks with a wide variety of ingredients [16,17] due to its advantage of generating products with taste, texture, size, and shape that are agreed upon by consumers [18]. Through extrusion, the raw ingredients are transformed into ready-to-eat products in an efficient, fast, and continuous way.

**Figure 1:**
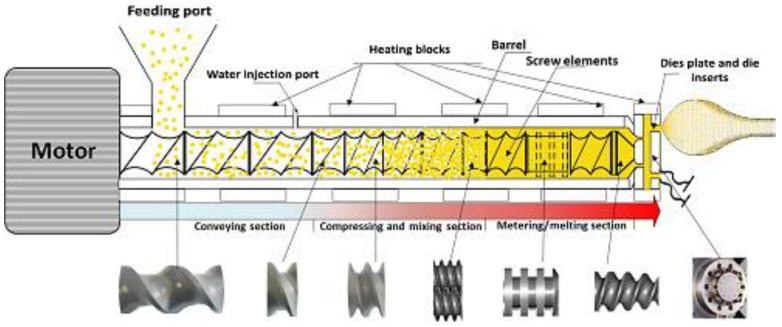
Extrusion processing of food ingredients, reprinted with permission from Ek and Ganjyal [15], published by Elsevier in 2020, copyright year 2023.

The equipment used for extrusion can have single- or twin-screws, and according to the thermal expenditure, it can be cold or thermoplastic, the one possessing two screws being more advantageous as it has a greater operational range which leads to better snack consistency and quality [19]. On the other hand, single-screw thermoplastic extruder is more common, with the barrel playing the role of a heat exchanger, which passes on the energy to the food matrix as it goes along the equipment [20]. The main components of an extruder include a “feeding hopper, barrel, screw(s), die, cutter, and the barrel encasing the screw(s) which are rotated by a motor”, and sometimes a preconditioner for material moisture adjustment [7] (Figure 1).

The extrusion processing consists of some “unit operations, such as mixing, shearing, starch gelatinization, protein denaturation, forming, texturizing, cooking, enzyme inactivation, cutting, puffing, and drying, which occur almost simultaneously” [7,21]. In extrusion, there are some input parameters such as material humidity, feeding rate, speed of the screw(s), temperature profile, die diameter, and length, while the system parameters refer to pressure, torque, and specific mechanical energy [15]. The output parameters include snack expansion properties, solubility, texture, chemical composition, and density [15]. Extrusion is a very versatile technique because the equipment can be set to operate in a vast range of conditions by changing the desired parameters, which will lead to different final product quality.

## 3. Bioactive Compounds

Phenolic compounds, vitamins, minerals, fibers, and terpenes are natural molecules found in legumes, fruits, cereals, and vegetables that contribute to human health due to their bio-functional properties, some of them contributing thus to the extension of food shelf-life. Thermal processing, including blanching, cooking, and autoclaving, is responsible for the decrease in bioactive compound content as a result of molecules leaching in the processing water [22]. On the other hand, extrusion is a processing technique with water restriction, so no effluents are implied, and thus, it is important to underline its effects on the bioactive compounds profile of the extruded final product. The main factors that determine the magnitude of changes in the bioactive compounds profile of foods are the ingredient’s nature, the processing technology, and time [22].

**Figure 2:**
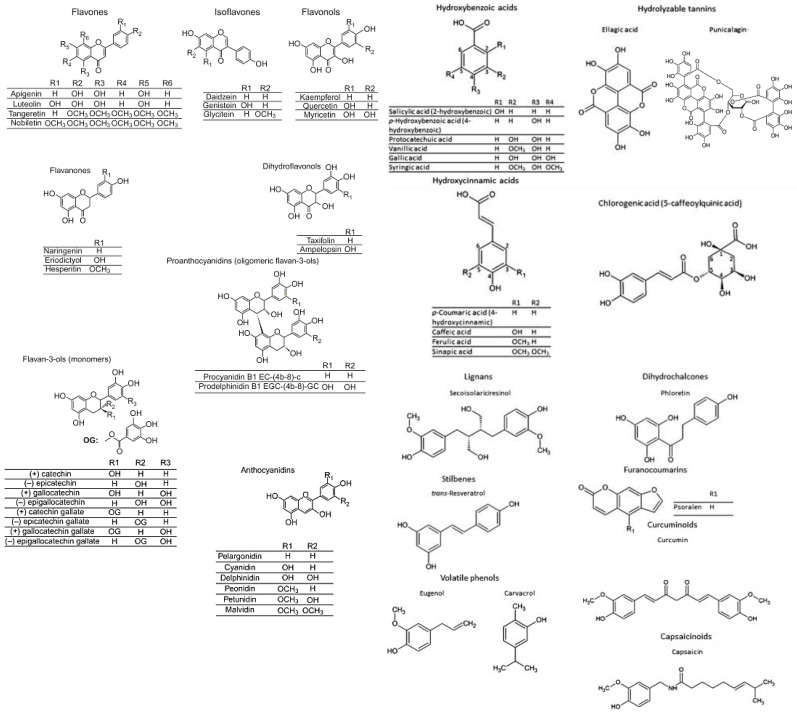
Structures of flavonoid, phenolic acids, and other non-flavonoid phenolic compounds reprinted with permission from de la Rosa et al. [23], published by Elsevier in 2019, copyright year 2023.

Phenolic compounds structure (Figure 2) comprises at least one aromatic ring with at least one hydroxyl group, which gives them great variability, being known more than 8000 phenolic structures in vegetal sources [23]. The phenols are considered natural secondary metabolites of plants, along with the isoprenoids [24]. The classification of phenolic compounds comprises flavonoids which are predominant in vegetal sources (flavones, isoflavone, flavonols, flavanones, dihydro flavonols, proanthocyanidins, flavan-3-ols, and anthocyanidins) and non-flavonoids such as phenolic acids (hydroxybenzoic acid, hydrolyzable tannins, hydroxycinnamic acids, and chlorogenic acid) and other phenolic compounds (lignans, dihydrochalcones, stilbenes, furanocoumarins, volatile phenols, curcuminoids, and capsaicinoids) [23]. In vegetal tissues, they can be linked to mono and polysaccharides and/or with esters and methyl esters [24].

The health benefits of the phenolic compounds from vegetal sources refer to antioxidant, anti-cancer, anti-inflammatory, anti-radical activity, and immune system boosting [25,26]. These properties of the phenolic compounds depend on their bioavailability which is strongly influenced by the nature and chemical structure of the molecule, the processing methods, the interactions that occur in the food matrix, and the body metabolism [26,27]. Phenolic compounds can remove the reactive oxygen species responsible for oxidative stress, prevent their formation by suppressing the activity of the enzymes implied in their formation, support the regeneration of the organism antioxidants (α-tocopherol and ascorbic acid), promote the adequate functioning of the signal transduction and cells antioxidant protection system [26,28]. Oxidative stress contributes to the development of neurodegenerative problems such as Alzheimer’s, which imply modifications of the cell DNA, fat, and protein fractions [26]. The intake of green tea phenolic compounds has been proven to diminish the accumulation of iron, determined fat peroxide, and other neurotoxic fat peroxides formed over time, reducing thus the risk of Parkinson’s appearance [29]. Yan et al. [30] summarized the effects of phenolic compounds from plants on the development and amelioration of neurodegenerative problems and concluded that these compounds exert neuroprotective action by their direct passing of the blood–brain barrier or by impacting the activity of intestinal microflora.

Regarding the anti-cancer activity of phenolic compounds, it was stated that they could diminish the metastasis of the cells in various ways, such as elimination of the carcinogenic compounds, regulation of cancer cell signaling and cell cycle progression, boosting of apoptosis and regulation of the enzymatic equipment [26,31]. Flavonoids were proven to reduce tumoral cell proliferation in various types of cancer, such as mammary cancer, melanoma, lung and liver, and digestive cancer [32]. The preventive role of phenolic compounds against cancer derives from their ability to prevent oxidation, reduce cell proliferation, promote organism detoxification, start apoptosis, stimulate the immune system, regulate hormonal activity, and inhibit inflammation [26,32]. It has been demonstrated that phenolic compounds exert antidiabetic activity that could be a result of the glucose absorption reduction promoted by them, stimulation of insulin and glucagon-like peptide 1 production, inhibition of glucose liberation from the liver and/or diminishing of the aldose reductase, α-amylase, and α-glucosidase enzymes activities [33,34]. The protective role of phenolic compounds on the cardiovascular system is related to their antioxidant capacity because lowering the low-density lipoprotein oxidative processes and the inflammatory response generates smaller blood pressure, enhanced endothelial cell functioning, and reduced platelets aggregation [35]. Phenolic compounds can change nitric oxide synthase enzyme activity and quantity, and modify the bioavailability of nitrogen monoxide (NO) for endothelium, a principle that forms the base mechanism action for cardiovascular health effects [35]. Polyphenols may exhibit anti-obesity effects by reducing weight, body mass index (BMI), waist circumference, and body fat mass, the main mechanisms associated being related to enzymatic activity, energy consumption, appetite diminishing, adipocyte discrimination, fat metabolism, and intestinal microflora activity [26,36]. The beneficial influence of phenolic compounds on human health depends on a series of factors such as the quantity ingested, the bioavailability, the body’s health status, sex, age, and living conditions.

**Figure 3:**
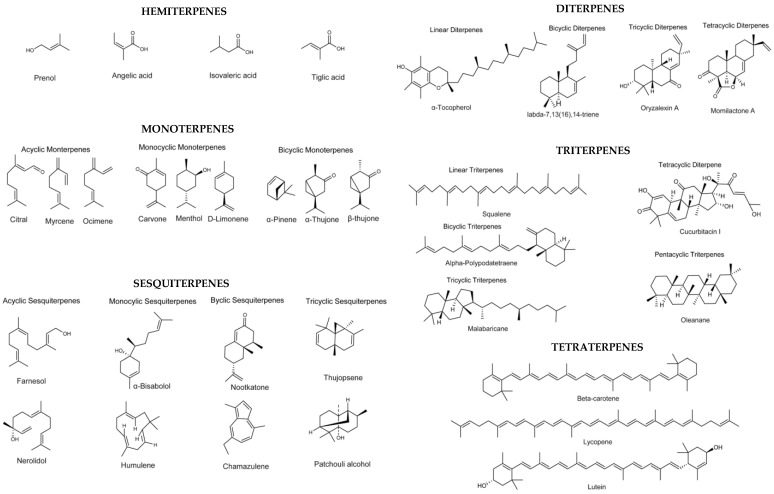
Structures of some terpenes found in vegetal sources, reprinted with permission from Ninkuu et al. [37], published by Elsevier in 2021, copyright year 2023.

Terpenes (Figure 3) are secondary metabolites found in vegetal sources formed of linear hydrocarbons or carbocyclic structures, with great variability (around 55,000 terpenes are known) [37]. The classification of terpenes is made in function of the number of isoprene groups (C_5_H_8_) which is the pillar of terpenes structure, while their transformation through oxygenation, hydrogenation, or dehydrogenation reactions give rise to terpenoids which are terpenes-like compounds [37]. Terpenes can be classified as “hemiterpenes (C_5_H_8_), monoterpenes (C_10_H_16_), sesquiterpenes (C_15_H_24_), diterpenes (C_20_H_32_), triterpenes (C_30_H_48_), tetraterpenes (C_40_H_64_), and polyterpenes (C_5_H_8_)” [38].

Terpenes and their glycosides were proven to contribute to the anti-inflammatory activity, oxidative stress reduction, antiaggregatory, anti-coagulative activity, anti-cancer, sedative, and analgesic ability of vegetal foods [39,40]. Terpene’s way of action in the human body includes interference with major molecular compounds, playing the role of immunostimulants, the modification of blood coagulation hemostasis, promotion of reactive oxygen species reduction, the modulation of transcription factors such as the nuclear factor kappa B (NF-κB) responsible for some processes in the inflammatory pathways leading to some illnesses such as cardiovascular problems, diabetes, Alzheimer, etc. [40,41]. Terpenes are also part of some vitamins like A, E, K, and coenzyme Q10, carotenoids (tetraterpenoids), while tocopherols are an important fount of vitamins A and E, respectively [40,42]. The data reported in the literature led to the consumption of carotenoids with health benefits, the ones comprising a minimum of one unsubstituted β-ring being precursors for retinol which is an essential micronutrient in the human diet. The consumption of products with raised carotenoid content may be related to cardiovascular problems reduction and the prevention of diseases such as diabetes (type II), obesity, and tumors [42].

**Figure 4:**
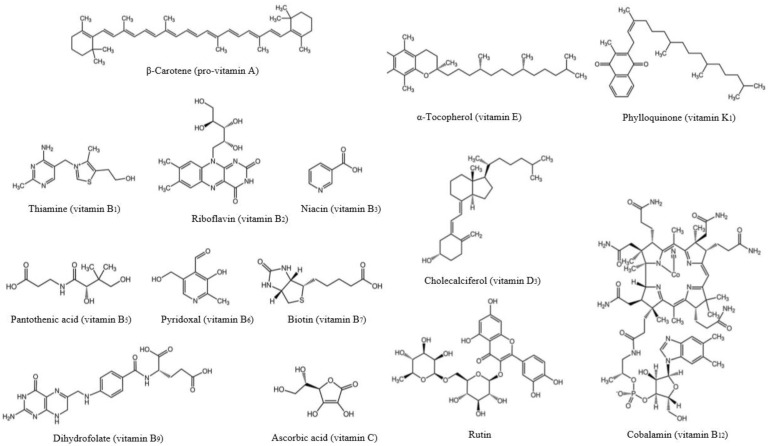
Structures of vitamins, reprinted with permission from Asensi-Fabado and Munne-Bosch [43], published by Elsevier in 2010, copyright year 2023.

Vitamins (Figure 4) are complex structural substances that the body is unable to produce, but they are mandatory for the accomplishment of certain of its fundamental functions [43].

Many vitamins function as such or after prior biotransformation, forming specific enzymes and coenzymes (all B vitamins, biotin, vitamins A and K). Others act on hormone-like pathways (D and A). Some (vitamins C and E) function as antioxidant systems against harmful peroxides. Vitamin deficiencies cause diseases that can be severe and even lethal in some cases. A deficiency of vitamins A, B1, B3, B6, C, and D can lead to significant health problems, such as blindness, beriberi, pellagra, anemia, scurvy, and rickets [44]. Vitamin E has antioxidant properties and protects unsaturated fatty acyl moieties of fat molecules from membranes [45]. Vitamins A (retinal, retinoic acid) have particular modes of action. An earlier classification divided vitamins into two major categories according to their structural properties as follows: the fat-soluble vitamins (A, D, E, and K) and the water-soluble vitamins (thiamin, riboflavin, niacin, pantothenic acid, vitamin B-6, folic acid, biotin, vitamin B-12, and vitamin C) [46]. The main characteristics of vitamin C and those of group B include a smaller retention by the body and a bigger elimination through urinary excretion [45]. They have complex biochemistry and are essential to human nutrition and health. Vitamins are important for human health since they are part of some enzyme structures implied in some biochemical and physiological activities in the body [44]. To maintain all the physiological and vital functions of the organism, the body needs vitamins and minerals, required in quantities of micrograms or milligrams per day [47]. Vitamin C (ascorbic acid) is involved in collagen production and acts as a cofactor in several crucial enzymatic responses, such as catecholamines, carnitine, and cholesterol synthesis, as well as in the activity of the transcription factors that manage the expression of important metabolic genes [48]. Additionally, it fights against oxidative stress and neurodegenerative problems or inflammation [49]. These highlights that vitamin C can contribute to preventing the circulatory system, chronic inflammation, and neurodegenerative conditions [49]. Vitamin E (tocopherol and tocotrienol) is present in the cellular walls and plasma lipoprotein and plays an important role in DNA, lipoproteins with low-density, and PUFA protection against oxidative stress. It takes part in hemoglobin formation, immune system functioning, and membrane structure stabilization [49]. Vitamin K1 is essential for blood coagulation, bone synthesis and repair, and a scarcity of vitamin K1 contributes to bleeding problems [50]. Vitamin E, which exerts high antioxidant activity, comprises compounds such as tocopherol and tocotrienols, molecules composed of a polar ring and a phytol chain of different sizes in function on the number of conjugated isoprenoid units [51].

Minerals are essential nutrients for organisms for growth and development and are divided into two groups: macro-minerals: calcium (Ca); magnesium (Mg); potassium (K); sodium (Na); chloride (Cl); phosphorus (P); and sulfur (S) and micro minerals: iodine (I); zinc (Zn); selenium (Se); iron (Fe); manganese (Mn); copper (Cu); cobalt (Co); molybdenum (Mo); fluoride (F); chromium (Cr); and boron (B). During the extrusion cooking, Ca, Mg, K, P, and Fe are the main components of the mineral profile investigated [46]. Fe is implied in O_2_ transport and transfer to the cells, and its deficiency can determine anemia [52]. Zn is part of some essential enzymes, and it is involved in protein formation, retardation, and biological processes deregulations occurring if it is deficient [52]. The main health functions of minerals are nervous system functioning and thyroid functioning (Mn), tooth and bone formation (P, Ca), fighting against infections and helping lung functions (Cu), heart regulation, and muscle relaxation (Mg), reducing blood pressure (K), nerve and muscle functioning (Na, K) [52].

Dietary fiber (DF) was defined for the first time in the 70 s as the remnants of edible plant cells, polysaccharides, lignin, and associated substances resistant to digestion by the alimentary enzymes of humans” [53]. Based on their water solubility, DF may be classified as soluble dietary fiber (SDF) and insoluble dietary fiber (IDF) [54]. The IDF is found in plants as a cell wall component, including cellulose, hemicellulose, and lignin, whereas SDF consists of a variety of non-cellulosic polysaccharides and oligosaccharides, such as pectins, water-soluble gum and β-glucans [55]. The consumption of dietary fiber is a key element of a healthy diet. A high-fiber diet protects against numerous chronic diseases, like obesity, heart disease, diabetes, colon cancer, kidney disease, and digestive problems, particularly irritable bowel syndrome [56]. Fibers play a key role in cleaning the body of toxins, helping to reduce inflammation and clear the arteries because they eliminate waste and pathogenic agents [57]. A diet enriched with functional fiber would be a new nutritional approach to prevent malnutrition-related illnesses [58]. Some extensive studies evaluated the association between fiber consumption and risk for colon or rectum cancer and found that people with lower fiber intake may present a higher risk [59]. Additionally, the literature highlights the effect of dietary fibers on the risk of cardiovascular problems [60]. High viscous fibers (such as oat glucans, pectins, and guar gum) influence blood lipid levels, while non-viscous fibers, such as wheat fiber and cellulose, typically do not [61]. Fiber is a major component of most plant food byproducts, particularly the nuts and seeds group. Dietary fiber use in humans has been linked to a lower risk for cardiovascular diseases and colorectal cancer [62,63].

### 3.1. Effects of Extrusion on the Phenolic Compounds and Terpenes

The impact of the extrusion process on the phenolic compounds and terpenes content depends on the processing conditions such as temperature, moisture, screw speed, and on the type of food matrix components (cereals, legumes, pseudocereals, vegetables, fruits, seeds, herbs or mixtures of them). Table 1 presents a synthesis of some results presented in the literature regarding the effect of extrusion on the total phenols and flavonoid content.

Song et al. [64] reported an increase in madecassoside, botulin, jasmone, and curcumol contents of quinoa flour after extrusion, while other phenolics like mangiferin and epigallocatechin were removed completely. The extrusion of maize determined the decrease of 28–35% of total phenols and of 30–37% of the flavonoid content, with bonded phenolics presenting the highest decrease compared to the raw material [65]. On the other hand, the bio-accessibility of total phenolics and flavonoids increased significantly after the extrusion of maize [65]. A study of the effects of extrusion on the properties of black and red rice revealed lower values of the total phenolic and flavonoid content compared to the raw materials due to the thermal damage of the phenolic compounds structures when exposed to high temperatures and/or the polymerization [66]. According to the data presented by Muñoz-Pabon et al. [67], the level of extractable phenolics decreased after the extrusion of quinoa flour, while the amount of hydrolyzable phenolics increased, which certified the leaching of phenolics that were bound to the cell walls. The extrusion of intermediate wheatgrass determined the rise of ferulic and p-coumaric acid content as a result of the free phenolics polymerization and/or bound phenolics release, while the sinapic acid decreased because it was found mostly in free form rather than bonded [68]. The investigation of extrusion effects on corn-quinoa and corn-amaranth properties revealed that the content of total phenols in amaranth-containing samples after acid-hydrolysis was higher after extrusion, while for those with quinoa, the total phenols content decreased [69]. The explanation for this behavior of the corn-amaranth sample would be that the bound phenolics were leached, and thus, they were better extracted for quantification, while in the case of quinoa, it might be possible that the protein complexation determined by their denaturation would dictate the decrease in total phenolics [69].

The extrusion of rice-cowpea-whey protein mixes determined the reduction in total polyphenols content due to the decomposition and/or destruction of the molecular structure of some phenolics at temperatures higher than 80 °C [70]. The extrusion of hemp seeds resulted in higher total phenolics, the number of free polyphenols being greater compared to the bound-ones, a fact that supports the hypothesis of the release of free phenols from the pericarp during extrusion which counteracted the losses caused by the oxidative damage [71]. The same study demonstrated that the screw speed determined a decrease in total phenols content due to the strong linkages through covalent and/or hydrogen bonds and hydrophobic interactions between complex flavonoids and fibers, compared to the non-flavonoid phenolic compounds [71]. Félix-Medina et al. [72] studied the effect of extrusion on maize-common bean mixes and found that free phenolics content was lowered, the most important compound identified being kaempferol hexoside. The main factors that influence phenolic compound quantity in extruded snacks are the moisture of the mix, temperature, and screw speed since they determine the magnitude of leaching and/or structure changes [72]. The study performed by Arribas et al. [22] showed that the extrusion of rice–carob–pea mixes lowered the amount of anthocyanins but increased the content of flavonols. Pea-rice mixes presented smaller content of total phenolics, while the samples containing carob, pea, and rice exhibited an opposite trend after extrusion, these differences being attributed to the distinct food matrix components since the carob ingredient is rich in fibers and thus the extrusion could have been promoted the release of phenolics [22].

The extrusion of corn-mango pomace mix caused the increase in low-molecular-weight compounds varieties, along with the decrease in “gallic acid-derived molecules, chlorogenic acids, maclurin-galloyl-glucoside, hepta-*O*-galloyl glucose, homo-mangiferin, isomanginferin, quercetin, and quercetin-3-*O*-glucoside” [73]. Extrusion promoted the breaking of high-molecular-weight compounds from corn-mango pomace mix (e.g., gallotannins like hexa-, hepta-, and octa-galloyl glucose), which resulted in raised phenolic acids and monomeric molecules [73]. The bio-accessibility of phenolic acids increased after extrusion, while that of xanthones and flavonoids decreased as a result of their capacity to form micelles soluble in water with fat fractions, non-polar micronutrients and other compounds from the digestive fluids [73]. According to the results obtained by Oladiran and Emmambux [74], the extrusion of cassava–soy–grape pomace mix determined the decrease in total phenols content due to the reduction in free phenolics amount and/or the degradation of phenolic molecules structured and polymerization reactions.

**Table 1:** Effects of extrusion on the phenolic compounds content.

Food Matrix	Experiment Conditions		Effect		Ref.
	Extrusion Parameters	Sample	Total Phenols	Total Flavonoid	
Rice with pea and carob fruit	- twin–screw extruder, medium rate of 25 kg/h, screw diameter of 25 mm, final barrel temperature of 125 °C, speed of 900–950 rpm; - water at a rate of 2.50 (sample without carob), 3.00 (sample with 5% carob), and 3.22 kg/h (sample with 10% carob).	- 20% pea 0% carob - 20% pea 5% carob - 20% pea 10% carob - 40% pea 0% carob - 40% pea 5% carob - 40% pea 10% carob	2.68 to 2.19 ^1^ ↓ 3.51 to 3.68 ^1^ ↑ 4.12 to 4.00 ^1^ ↓ 3.46 to 3.21 ^1^ ↓ 3.53 to 4.95 ^1^ ↑ 3.89 to 5.55 ^1^ ↑	0.03 to 0.02 ^2^ ↓ 0.04 to 0.05 ^2^ ↑ 0.08 to 0.06 ^2^ ↓ 0.01 to 0.03 ^2^ ↑ 0.02 to 0.06 ^2^ ↑ 0.04 to 0.08 ^2^ ↑	[22]
Corn grits with turmeric, ginger, bay leaf, or laurel	- 13% moisture, temperature of 180 °C, screw speed of 700 rpm, 3 mm diameter die, feeding rate 3 kg/min.	- control (corn) - 3% laurel - 3% turmeric - 3% ginger - 3% mixture (1:1:1)	180.37 to 21.63 ^3^ ↓ 214.55 to 119.38 ^3^ ↓ 212.65 to 182.32 ^3^ ↓ 246.98 to 125.92 ^3^ ↓ 224.72 to 139.66 ^3^ ↓	57.79 to 76.6 ^2^ ↑ 205.75 to 221.03 ^2^ ↑ 282.31 to 141.00 ^2^ ↓ 250.87 to 90.10 ^2^ ↓ 246.31 to 103.68 ^2^ ↓	[75]
Maize and bean	- single-screw laboratory extruder, 19 mm diameter screw, 3 mm die diameter, barrel temperature of 164 °C, screw speed of 187 rpm.	- 70% maize + 30% bean flour	260.83 to 256.69 ^3^ ↓	87.37 to 86.23 ^3^ ↓	[72]
Pearl millet with almond cake	- twin-screw extruder, with circular die of 3 mm diameter, temperature of 60 °C in the first step and 80 °C in the second one, 120 °C for the last step, 450 rpm speed.	- 80% pearl millet + 20% almond cake	60.34 to 56.91 ^3^ ↓	20.65 to 18.29 ^3^ ↓	[76]
Rice	- co-rotating twin-screw extruder, 15.5% and 16% of feed moisture, 159 and 150 °C for the 4th barrel zone temperature for black and red rice, respectively.	- black rice - red rice	569.29 to 180.71 ^4^ ↓ 425.74 to 100.21 ^4^ ↓	496.89 to 153.71 ^1^ ↓ 280.40 to 99.10 ^1^ ↓	[66]
Chokeberry pomace powder	- co-rotating twin-screw extruder, screw diameter of 25.5 mm, 7 sections, feedingJuly 2023 rate of 9 and 8 kg/h, 13% and 23% moisture, die of 3 mm diameter, temperature steps of 40/60/80/100/100/100 °C.	- 200 rpm, 13% moisture - 400 rpm, 13% moisture - 800 rpm, 13% moisture - 800 rpm, 23% moisture	55.00 to 62.00 ^1^ ↑ 55.00 to 67.00 ^1^ ↑ 55.00 to 73.00 ^1^ ↑ 55.00 to 64.00 ^1^ ↑	18.00 to 6.10 ^1^ ↓ 18.00 to 4.30 ^1^ ↓ 18.00 to 2.40 ^1^ ↓ 18.00 to 5.80 ^1^ ↓	[77]
Maize	- single-screw extruder, zones temperatures of 60/70/80/−90 °C, die diameter of 5 mm, screw speed of 60 rpm.	- 20% moisture - 25% moisture - 30% moisture	129.60 to 95.20 ^4^ ↓ 129.60 to 88.50 ^4^ ↓ 129.60 to 97.90 ^4^ ↓	0.11 to 0.08 ^1^ ↓ 0.11 to 0.07 ^1^ ↓ 0.11 to 0.07 ^1^ ↓	[65]
Mulberry varieties leaf	- tween-screw extruder, barrel temperature of 100 °C, pressure of 40~50 bar, screw speed of 50 rpm	- Cheongol - Iksu - Cheongil	14.48 to 31.14 ^5^ ↑ 11.96 to 15.72 ^5^ ↑ 11.17 to 23.96 ^5^ ↑	8820 to 19,680 ^2^ ↑ 8770 to 22,120 ^2^ ↑ 12,260 to 22,020 ^2^ ↑	[78]
Corn with defatted soy and spinach	- single-screw extruder, screw speed of 140 rpm, die diameter of 2 mm, temperature profile of 120/150/170/150 °C, feed rate of 300 g/ min	- Control (corn) - 40% soy + 10% spinach - 30% soy + 20% spinach - 20% soy + 30% spinach - 10% soy + 40% spinach	1.16 to 0.93 ^1^ ↓ 3.36 to 2.68 ^1^ ↓ 4.52 to 3.61 ^1^ ↓ 5.30 to 4.24 ^1^ ↓ 6.24 to 4.99 ^1^ ↓	0.58 to 0.47 ^1^ ↓ 1.23 to 0.98 ^1^ ↓ 1.75 to 1.40 ^1^ ↓ 2.26 to 1.80 ^1^ ↓ 2.58 to 2.06 ^1^ ↓	[79]
Fenugreek seed and leaf, oat, pea, rice, and corn	- twin-screw extruder, feed moisture content of 12%, barrel temperatures of 110 °C, screw speed 200 rpm.	- 22% oat flour + 9% green pea flour + 2% fenugreek seed + fenugreek leaf flour 0.70% + 53.04% rice + 13.26% corn;	-	1.00 to 0.80 ^1^ ↓	[80]
Mustard meal concentrate	- twin-screw extruder, barrel temperature of 100–150 °C, screw speed 250–350 rpm, moisture of 12–18%.	- 5% mustard meal - 10% mustard meal - 15% mustard meal	76.47 to 75.11 ^3^ ↓ 84.49 to 82.63 ^3^ ↓ 110.76 to 108.54 ^3^ ↓	-	[81]
Cassava-soy composite with grape pomace	- co-rotating twin-screw extruder, 5 heating areas at 60/80/100/140/140 °C, water 3 L/h, feed rate of 25 kg/h, die diameter of 3 mm, screw speed of 200 rpm.	- 0% grape pomace - 10% grape pomace - 20% grape pomace	2.1 to 0.9 ^1^ ↓ 5.5 to 3.5 ^1^ ↓ 7.7 to 5.9 ^1^ ↓	-	[74]
Corn flour with pea protein, broccoli, lucerne, beetroot, rosehip, turmeric, chili, paprika, and basil	- single-screw laboratory extruder, screw diameter of 19 mm, 4 heating areas (50/100/140/140 °C), die diameter of 4 mm, screw speed of 100 rpm, and feeding speed of 20 rpm;	- Control (corn) - 2% pea - 5% broccoli - 5% lucerne - 15% beetroot - 15% rosehip - 2% chili - 2% turmeric - 2% paprika - 2% basil	95.23 to 55.78 ^1^ ↓ 98.62 to 72.97 ^1^ ↓ 122.96 to 94.73 ^1^ ↓ 100.77 to 81.17 ^1^ ↓ 213.01 to 172.29 ^1^ ↓ 260.30 to 312.02 ^1^ ↑ 98.05 to 80.05 ^1^ ↓ 104.11 to 77.49 ^1^ ↓ 99.15 to 74.81 ^1^ ↓ 117.85 to 104.16 ^1^ ↓	-	[82]
Corn with brewer’s spent grain, sugar beet pulp, apple pomace	- blends with 15% moisture content; - single-screw extruder, temperature steps of 135/170/170 °C, compression ratio of 4:1, die of 4 mm diameter.	- Corn grits - 5% brewer’s spent grain - 10% brewer’s spent grain - 15% brewer’s spent grain - 5% sugar beet pulp - 10% sugar beet pulp - 15% sugar beet pulp - 5% apple pomace - 10% apple pomace - 15% apple pomace	61.38 to 48.39 ^3^ ↓ 65.32 to 53.32 ^3^ ↓ 69.74 to 60.10 ^3^ ↓ 71.20 to 63.38 ^3^ ↓ 53.76 to 51.33 ^3^ ↓ 53.30 to 51.04 ^3^ ↓ 51.00 to 94.90 ^3^ ↓ 240.37 to 167.18 ^3^ ↓ 337.86 to 285.36 ^3^ ↓ 421.09 to 409.13 ^3^ ↓	-	[83]
Corn, carrot powder, ascorbic acid	- single-screw extruder, temperature profiles of 135/170/170 °C, 4:1 compression ratio screw, screw speed of 100 rpm, feed rate of 15 rpm.	- control - 4% carrot powder - 6% carrot powder - 8% carrot powder	0.73 to 0.51 ^5^ ↓ 0.75 to 0.70 ^5^ ↓ 0.82 to 0.75 ^5^ ↓ 1.01 to 0.87 ^5^ ↓	-	[84]
Quinoa flour	- moisture adjusted at 18%; - twin-screw extruder, temperature of 75 °C, 105 °C, and 135 °C, screw rotation speed of 251–253 rpm, three nozzles of 2.6 mm diameter.	- quinoa flour	1.79 to 0.75 ^5^ ↓	-	[67]
Corn with pea and rosehip	- single-screw laboratory extruder, barrel diameter of 19 mm, 3:1 compression ratio, dosing speed of 18 rpm, feed rate of 3.51 kg/h, rotation speed of 150 rpm, temperatures of barrel sections of 25/70/170/175 °C, nozzle diameter of 3 mm.	- control (corn flour) - 10% rosehip; - 10% rosehip + pea protein	23.68 to 14.40 ^3^ ↓ 334.00 to 169.20 ^3^ ↓ 272.00 to 169.30 ^3^ ↓	-	[85]
Corn grits with germinated and dehulled chickpea, tomato powder, skim milk	- single–screw extruder temperatures steps at 100/160/180 °C, respectively, feeding screw speed 160 rpm, barrel screw speed 250 rpm, screw compression 4:1, die diameter of 3 mm.	- control - 10% chickpea - 20% chickpea - 30 % chickpea	7.17 to 7.35 ^5^ ↑ 9.20 to 3.37 ^5^ ↑ 9.76 to 10.38 ^5^ ↑ 10.30 to 11.11 ^5^ ↑		[86]
Corn grits with cocoa husk	- single-screw extruder, 4:1 screw, die diameter of 4 mm, temperature profile: 135/170/170 °C.	- control - 5% cocoa husk - 10% cocoa husk - 15% cocoa husk	55.17 to 48.63 ^3^ ↓ 84.37 to 72.25 ^3^ ↓ 105.14 to 83.99 ^3^ ↓ 109.91 to 105.68 ^3^ ↓	-	[87]
Corn grits with carrot pulp	- twin-screw extrudeJuly 2023r, die diameter of 3 mm, feeding rate of 36 ± 1 g/min, temperature profile 1 (80/90/100/130/120 °C) and 2 (80/105/130/160/130 °C), screw speed of 125 or 225 rpm.	- temperature profile 1, 125 rpm - temperature profile 1, 225 rpm - temperature profile 2, 125 rpm - temperature profile 1, 225 rpm	18.15 to 10.26 ^5^ ↓ 18.15 to 9.45 ^5^ ↓ 18.15 to 9.60 ^5^ ↓ 18.15 to 10.01 ^5^ ↓	-	[88]
Corn grits with tomato powder	- single-screw polytrophic extruder, screw compression ratio of 3:1, barrel temperatures of 125–145–135 °C, die diameter of 3 mm, screw speed of 120 rpm.	- Control - 5% tomato - 10% tomato - 15% tomato - 20% tomato - 25% tomato - 30% tomato	67.1 to 48.8 ^3^ ↓ 83.8 to 107.4 ^3^ ↑ 84.2 to 173.9 ^3^ ↑ 96.3 to 202.7 ^3^ ↑ 98.6 to 214.5 ^3^ ↑ 103.1 to 223.3 ^3^ ↑ 109.7 to 239.6 ^3^ ↑	-	[89]
Lupin seed coat	- co-rotating intermeshing twin-screw extruder, feed rate of 4 kg/h, variable barrel temperatures (120–150 °C), screw speed of 400 rpm, moisture of 40%.	- lupin seed coat at 120 °C - lupin seed coat at 135 °C - lupin seed coat at 150 °C	54.26 to 47.55 ^3^ ↓ 54.26 to 50.32 ^3^ ↓ 54.26 to 46.65 ^3^ ↓	-	[90]
Soybean, canola, sunflower cakes	- twin-screw extruder, temperature profile of 40/60/80/100/130 °C moisture of 17%, feeding rate of 13.2 Kg/h, screw speed of 500 rpm.	- soybean cake - canola cake - sunflower cake	2.5 to 2.6 ^5^ ↑ 35.7 to 43.1 ^5^ ↑ 25.4 to 8.2 ^5^ ↓	-	[91]
Nut shell	- twin-screw co-rotating extruder, feed rate of 7.79 kg/h d.m., screw speed of 100–200 rpm, barrel temperature of 33.25–106.75 °C.	- nut shell at 100 rpm, 40 °C - nut shell at 100 rpm, 100 °C - nut shell at 150 rpm, 33.25 °C - nut shell at 150 rpm, 70 °C - nut shell at 150 rpm, 106.75 °C - nut shell at 200 rpm, 40 °C - nut shell at 200 rpm, 100 °C	36.14 to 32.82 ^5^ ↓ 36.14 to 34.48 ^5^ ↓ 36.14 to 21.29 ^5^ ↓ 36.14 to 69.83 ^5^ ↑ 36.14 to 37.04 ^5^ ↑ 36.14 to 34.98 ^5^ ↓ 36.14 to 37.34 ^5^ ↑	-	[92]

^1^—expressed as mg CE (catechin equivalent)/g d.w., ^2^—expressed as μg QE (querciton equivalent)/g d.w., ^3^—expressed as mg GAE (gallic acid equivalent)/100 g, ^4^—expressed as mg FAE (ferulic acid equivalent)/100 g d.w., ^5^—expressed as mg GAE/g, ↑ increase, ↓ decrease.

Similar results were obtained by Jozinović et al. [83], which demonstrated a reduction in the total phenolic content of corn snacks with brewer’s spent grain, sugar beet pulp, or apple pomace addition after extrusion. Schmid et al. [93] reported that the total phenolic acids content, the total flavonols, quercitin glycosides, and the 3- and 5-caffeoylquinic acid isomers were not significantly affected by the extrusion of corn-chokeberry pomace flours, while the 4-caffeoylquinic acid content increased as a result of isomerization promoted by heat. The authors reported differences regarding the total content of phenols measured by the Folin–Ciocalteu method, which remained unchanged after extrusion, and the one calculated by summing the anthocyanins, phenolic acids, and flavonols contents determined by HPLC—value that decreased after extrusion. In contrast to other studies, Wang et al. [94] stated that there are no significant differences in total phenolic content of corn starch-cherry pomace mixes after extrusion, probably due to the protection role of starch and/or to the short residence time of extrusion. The particle size of the pomace added also influenced the number of total phenolics of the extrudates, with smaller particle size resulting in greater total phenolics content, a fact that could be attributed to a better opportunity for the starch matrix to protect the phenolics [94].

The improvement in corn flour with lucerne resulted in higher phenolics content, while the extrusion caused some changes in the phenolic profile of the mixtures, as follows: “Di-caff acid and Fer phenolic acid raised after extrusion, apigenin-glucoside, genistein, apigenin-glucuronide, and apigenin-diglucuronide were completely lost” [95]. There are some possible explanations for the loss or reduction in flavones and isoflavones content after extrusion of corn–lucerne mixes: either polymerization reactions and/or damage of the heat-sensitive phenolic structures occurred, or the heat affected the phenolic acid oxidation process, or the denatured-protein phenolic complexes were formed during extrusion which led to lower phenolics release [69,96]. Amer and Rizk [75] reported changes in phenolic compound levels in maize snacks fortified with ginger, bay leaves, and turmeric flours without major changes in the phenolic profile. The most abundant compounds found in extrudates were ferulic acid and catechin, the addition of herbs led to the rise in phenolic compounds content, especially gingerol in ginger-containing snacks and curcumin in turmeric-containing samples, respectively [75]. The results obtained by Culeţu et al. [82] for corn extrudates enriched with pea protein, broccoli, lucerne, beetroot, turmeric, chili, paprika, and basil revealed that extrusion reduced the total phenolic content by 11 to 41%, depending on the sample formulation, with the lowest impact observed on the basil-containing snack and the highest on the control. However, the same study demonstrated that the corn–rosehip sample exhibited an opposite trend, so the total phenolics increased after processing [82].

The impact of extrusion on the content of terpenes and terpenoid compounds in vegetal food matrices reported in the literature is presented in Table 2. Cueto et al. [97] reported a decrease in the carotenoid content of maize-based extrudates with quinoa or chia addition, the highest impact being observed for the chia-containing sample. The extrusion promotes the lipids leaching from the cells and thus contributes to greater exposure to carotenoids [97]. The significant decrease in carotenoid content in the corn-chia mix could be explained by the high content of unsaturated fat that can be oxidized during extrusion and/or can contribute to the formation of peroxides and free radicals able to react with carotenoids [97]. The carotenoid degradation could be diminished by using high extrusion moisture, temperature, and viscosity, a fact supported by the results obtained by Ortiz et al. [98] for biofortified maize. The loss of *cis*-isomers of β-carotene and other carotenoids during extrusion was attributed to the effect of temperature rather than the moisture of the sample, the effects of extrusion on the carotenoid retention being similar regardless of their molecular structure [98]. The extrusion of maize determined the reduction of lutein, zeaxanthin, and β-carotene by 53–63%, by 69–75%, and by 31–61%, respectively, in the function of the moisture of the sample [65]. These results could be attributed to the low heat stability of these compounds, isomerization, and/or the mechanical stress induced by the shear force and pressure [65]. The same study reported no significant differences regarding the content of campesterol, stigmasterol, and β-sitosterol after extrusion of maize, except for the sample with 20% moisture which exhibited lower values and raised bio-accessibility of them, a fact that could be due to the effect of temperature and form (free or bound) of phytosterol [65]. Boakye et el. [68] observed a decrease in lutein and zeaxanthin retention after the extrusion of intermediate wheatgrass, with the screw speed being a key factor in establishing the magnitude of losses since, at higher speeds, the degradation was less intense because of the enhanced extractability of carotenoids from the epicarp fraction under the shear force action. Zeaxanthin was found in greater amounts in the bran compared to the refined flour, while lutein was more present in the endosperm. The carotenoids from bran were proved to be less affected by extrusion, and thus, it can be explained by the more intense loss of lutein compared to zeaxanthin [68]. The use of higher extrusion temperatures resulted in greater β-carotene losses in corn–carrot pomace mixes, while lutein was reduced to the same extent regardless of the temperature profile, as demonstrated by Ortak et al. [88]. The decrease in β-carotene content could be explained by its isomerization from trans to cis form, which is more soluble, being known that in its natural state, β-carotene from carrot is mainly found in trans configuration [88].

**Table 2:** Effects of extrusion on the terpene and terpenoid content.

Food Matrix	Experiment Conditions		Terpenes		Ref.
	Extrusion Parameters	Sample	Type	Amount	
Corn, carrot powder, ascorbic acid	- single-screw extruder, temperature profiles of 135/170/170 °C, 4:1 compression ratio screw, screw speed of 100 rpm, feed rate of 15 rpm.	- control - 4% carrot powder - 6% carrot powder - 8% carrot powder	9-*cis*-β-Carotene (mg EβC/kg)	0.00 to 0.00 = 0.52 to 0.00 ↓ 0.44 to 0.44 = 0.74 to 0.79 ↑	[84]
		- control - 4% carrot powder - 6% carrot powder - 8% carrot powder	Lutein (mg/kg)	32.80 to 87.70 ↑ 21.70 to 36.70 ↑ 21.20 to 52.00 ↑ 16.70 to 39.90 ↑	
		- control - 4% carrot powder - 6% carrot powder - 8% carrot powder	Zeaxanthin (mg/kg)	32.80 to 42.90 ↑ 25.30 to 29.00 ↑ 19.10 to 24.40 ↑ 25.90 to 25.80 ↓	
Quinoa flour	- moisture adjusted at 18%; - twin-screw extruder, temperature of 75 °C, 105 °C, and 135 °C, screw rotation speed of 251–253 rpm, three nozzles of 2.6 mm diameter.	- quinoa flour	Total carotenoids (mg β-carotene/g d.w.)	11.33 to 8.39 ↓	[67]
Corn with pea and rosehip	- single-screw laboratory extruder, barrel diameter of 19 mm, 3:1 compression ratio, dosing speed of 18 rpm, feed rate of 3.51 kg/h, rotation speed of 150 rpm, temperatures of barrel sections of 25/70/170/175 °C, nozzle diameter of 3 mm.	- control (corn flour) - 10% rosehip; - 10% rosehip + pea protein	Total carotenoids (mg β-carotene/100 g d.w.)	3.75 to 2.11 ↓ 43.74 to 7.85 ↓ 22.73 to 5.76 ↓	[85]
Corn with carrot pomace	- co-rotating twin-screw extruder, screw diameter of 20 mm, 15% moisture of corn-carrot pomace mix, 20 mm 4 individual heating zones (50/100/140/140 °C), die with a diameter of 4.0 mm.	- 5% carrot pomace - 10% carrot pomace - 15% carrot pomace	β-carotene (mg/100 g)	0.34 to 0.26 ↓ 0.53 to 0.29 ↓ 0.96 to 0.34 ↓	[99]
Maize	- single-screw extruder, zones temperatures of 60/70/80/90 °C, die diameter of 5 mm, screw speed of 60 rpm.	- 20% moisture - 25% moisture - 30% moisture	Lutein (μg/100 g)	94.30 to 39.50 ↓ 94.30 to 43.0 ↓ 94.30 to 34.0 ↓	[65]
		- 20% moisture - 25% moisture - 30% moisture	Zeaxanthin (μg/100 g)	189.70 to 61.0 ↓ 189.70 to 54.00 ↓ 189.70 to 47.70 ↓	
		- 20% moisture - 25% moisture - 30% moisture	β-carotene (μg/100 g)	15.90 to 11.30 ↓ 15.90 to 8.30 ↓ 15.90 to 6.40 ↓	
		- 20% moisture - 25% moisture - 30% moisture	Campesterol (mg/100 g)	23.30 to 21.20 ↓ 23.30 to 22.40 ↓ 23.30 to 26.30 ↓	
		- 20% moisture - 25% moisture - 30% moisture	Stigmasterol (mg/100 g)	13.70 to 12.90 ↓ 13.70 to 14.00 ↓ 13.70 to 15.60 ↓	
		- 20% moisture - 25% moisture - 30% moisture	β-sitosterol (mg/100 g)	140.30 to 121.60 ↓ 140.30 to 127.70 ↓ 140.30 to 145.90 ↓	
Corn grits with carrot	- twin-screw extruder, die diameter of 3 mm, feeding rate of 36 ± 1 g/min, temperature profile of 80/90/100/130//120 °C, screw speed of 125 rpm	- corn + carrot pulp	β-carotene (μg/g d.w.)	11.13 to 4.23 ↓	[88]
			Lutein (μg/g d.w.)	2.66 to 1.91 ↓	
Intermediate wheatgrass	- corotating twin-screw extruder, screw diameter of 20 mm, temperature profile of 50/80/120/150/150/150 °C, screw speed of 200 rpm.	- intermediate wheatgrass	Lutein (μg/g flour)	14.53 to 3.52 ↓	[68]
			Zeaxanthin (μg/g flour)	3.39 to 1.16 ↓	
Maize	- co-rotating twin-screw extruder, barrel temperature of 140.7 °C, feed moisture of 20%, screw speed of 400 rpm, 5 mm die diameter	- PVAH 79–100 ^1^ - PVAH 1–26 ^1^ - PVAH 27–49 ^1^ - PVAH 50–75 ^1^	β-Carotene (mg/kg)	0.83 to 0.49 ↓ 0.83 to 0.41 ↓ 0.86 to 0.47 ↓ 0.88 to 0.49 ↓	[100]
		- PVAH 79–100 ^1^ - PVAH 1–26 ^1^ - PVAH 27–49 ^1^ - PVAH 50–75 ^1^	9-*cis*-β-Carotene (mg/kg)	0.53 to 0.36 ↓ 0.52 to 0.38 ↓ 0.56 to 0.41 ↓ 0.57 to 0.36 ↓	
		- PVAH 79–100 ^1^ - PVAH 1–26 ^1^ - PVAH 27–49 ^1^ - PVAH 50–75 ^1^	13-*cis*-β-Carotene (mg/kg)	0.47 to 0.33 ↓ 0.47 to 0.31 ↓ 0.47 to 0.32 ↓ 0.47 to 0.33 ↓	
		- PVAH 79–100 ^1^ - PVAH 1–26 1PVAH 27–49 ^1^ - PVAH 50–75 ^1^	β-Cryptoxanthin (mg/kg)	1.17 to 0.39 ↓ 1.53 to 0.39 ↓ 1.15 to 0.39 ↓ 1.21 to 1.38 ↓	
Corn with peach palm	- twin-screw extruder, temperature profile of 30/50/80/90/90/100/100/100/130/130 °C, screw speed of 400 rpm, die diameter of 8 mm, flow rate of 8 kg/h, moisture of 16 g/100 g	- 100 % corn - 15% yellow peach palm - 25% yellow peach palm - 15% red peach palm - 15% red peach palm	Lutein (μg/g d.w.)	0.78 to 1.72 ↑ 0.55 to 1.23 ↑ 1.25 to 0.80 ↓ 0.60 to 1.34 ↑ 0.55 to 0.58 ↑	[101]
		- 100 % corn - 15% yellow peach palm - 25% yellow peach palm - 15% red peach palm - 15% red peach palm	Zeaxanthin (μg/g d.w.)	1.11 to 3.39 ↑ 0.57 to 1.18 ↑ 1.32 to 1.64 ↑ 1.24 to 1.52 ↑ 0.73 to 0.94 ↑	
		- 100 % corn - 15% yellow peach palm - 25% yellow peach palm - 15% red peach palm - 15% red peach palm	β-cryptoxanthin (μg/g d.w.)	0.42 to 0.78 ↑ 0.50 to 0.52 ↑ 0.36 to 0.47 ↑ 0.45 to 0.58 ↑ 0.22 to 0.47 ↑	
		- 100 % corn - 15% yellow peach palm - 25% yellow peach palm - 15% red peach palm - 15% red peach palm	α-carotene (μg/g d.w.)	0.02 to 0.00 ↓ 0.10 to 0.09 ↓ 0.15 to 0.12 ↓ 0.15 to 0.14 ↓ 0.12 to 0.25 ↑	
		- 100 % corn - 15% yellow peach palm - 25% yellow peach palm - 15% red peach palm - 15% red peach palm	β-carotene (μg/g d.w.)	0.44 to 0.68 ↑ 1.33 to 0.84 ↓ 1.62 to 1.28 ↓ 1.91 to 1.77 ↓ 1.38 to 2.50 ↑	
		- 100 % corn - 15% yellow peach palm - 25% yellow peach palm - 15% red peach palm - 15% red peach palm	13-*cis* β-carotene (μg/g d.w.)	0.09 to 0.20 ↑ 0.20 to 0.15 ↓ 0.19 to 0.19 = 0.27 to 0.37 ↑ 0.21 to 0.53 ↑	
		- 100 % corn - 15% yellow peach palm - 25% yellow peach palm - 15% red peach palm - 15% red peach palm	9-*cis* β-carotene (μg/g d.w.)	0.10 to 0.29 ↑ 0.21 to 0.21 = 0.25 to 0.28 ↑ 0.34 to 0.46 ↑ 0.22 to 0.48 ↑	
Corn grits with tomato powder	- single-screw polytrophic extruder, screw compression ratio of 3:1, barrel temperatures of 125–145–135 °C, die diameter of 3 mm, screw speed of 120 rpm.	- Control - 5% tomato - 10% tomato - 15% tomato - 20% tomato - 25% tomato - 30% tomato	Lycopene (mg/100 g)	0.2 to 0.2 = 4.6 to 0.4 ↓ 9.7 to 1.6 ↓ 15.7 to 4.1 ↓ 21.4 to 7.8 ↓ 25.2 to 17.9 ↓ 31.2 to 22.3 ↓	[89]

^1^—maize variety, = unchanged, ↑ increase, ↓ decrease.

On the contrary, Obradović et al. [84] obtained higher values of lutein, zeaxanthin, and 9-*cis*-β-carotene after extrusion of corn–carrot mixes as a result of molecular structure changes induced by heat and pressure, which promoted better extractability. The differences between the effects of extrusion on the carotenoid contents presented in the literature could be due to the different operating conditions of the extruder, the food matrix, and the processing methodology (for example, Obradović et al. used ascorbic acid in the formulation which exerted a stabilization effect on the β-carotene). Basto et al. [101] also reported higher total carotenoid content of corn–peach palm mixes after extrusion, the magnitude of changes depending on the peach palm variety and addition level. Some explanations for the enhancement and preservation of β-carotene after processing could be the protective role of the protein-carbohydrate matrix, the short processing time which limited the destruction of the low-molecular weights compounds, and/or the breaking of cell walls under thermal and shearing forces which led to greater carotenoids extraction yield [101].

The literature reported different results regarding the impact of extrusion on the phenolics and terpenes profiles of food matrices from vegetal sources. These differences are mainly due to the characteristics of each formulation, the extrusion conditions, and the analytical method used for phenols quantification.

### 3.2. Effects of Extrusion on Vitamins and Minerals Content

The effects of extrusion on vitamins and minerals (Table 3) vary due because of the large diversity of their chemical structures. Vitamins A and E are the most unstable fat-soluble vitamins in comparison with vitamins D and K [102]. Low moisture and high-temperature speed up the carrot pomace products extrusion and highlight a decrease in β-carotene and vitamin C levels when the temperature increases [103]. A decrease of about 63% of vitamin E from buckwheat for all the temperatures used was also reported in another paper, a fact that is widely attributed to the loss of γ-tocopherol [104]. From the B-group, thiamine and riboflavin present a high research interest after extrusion cooking. The literature revealed a sensitivity of thiamine to heat as compared to riboflavin. Higher feed moisture and lower temperature typically result in increased retention of this vitamin [105]. An explanation can be the reduced viscosity at high moisture levels, increasing the material flow and reducing the dwell time in the barrel. Another research found that vitamin B12 stability in puffed snacks was completely destroyed at 194 °C, whereas when the feed rate and screw speed were changed, the stability was retained [106]. A high decrease in vitamin E (78%) was found in corn, wheat, soybean, and lentil flour when mild conditions were applied (high moisture content and low temperature) [107]. This fact indicates that the extrusion temperature has a very large impact on tocopherol, even if the exposure time is reduced. An increasing trend in the stability of the total content of tocotrienols during extrusion was observed in other research papers carried out on rice, buckwheat, amaranth, and quinoa [108,109]. This rise was explained by the increased stability and better extraction during food processing [102]. Additionally, vitamin A presents a high sensitivity during the extrusion process, which can be reduced with a short time exposure, increasing the screw speed and the form of the pro-vitamin. For example, carotenoid destruction in corn was diminished when quinoa or chia flour was added as a result of their great unsaturated fat amount [97]. The extrusion process can impact the vitamin content, and the most critical factor is the processing temperature of the extrusion.

Scarce research papers looked at the effects of extrusion on minerals, this fact being due to their stability under most food processing conditions. As against temperature, the high feed moisture seems to have a greater influence on the decrease in complexed iron content [110]. Instead of being released from complexes during the processing, a part of the complexed iron was oxidized during extrusion, explaining its decrease [110]. On the contrary to vitamins, even if extrusion does not affect mineral stability, their bioavailability can be modified [46]. Inconsistent results were found concerning the effect of extrusion on mineral bioavailability. Contradictory findings reported by various researchers for mineral content can be explained by the different techniques employed for determining bioaccessibility or as a result of the different chemical compositions of the food matrix [111]. A high Fe bioaccessibility after the extrusion process was observed for maize, sorghum, legumes, and dry beans [111,112], while for whole grain red sorghum, only Zn bioaccessibility was affected and Fe was not [113]. The main factor affecting minerals’ bioavailability after extrusion is the change of their binding to other components like phytic acid, phenolic compounds, dietary fibers, and proteins [114].

**Table 3:** Effects of extrusion on the vitamins and minerals content.

Food Matrix	Experiment Conditions		Vitamin	Effect	Ref.
	Extrusion Parameters	Sample			
Pea	- single-screw laboratory extruder, screw speed 60 rpm, barrel temperature 129 °C, 40, 34, 25 L/h moisture.	- Extruded at 129 °C - Extruded at 135 °C - Extruded at 142 °C	Thiamine (mg/100 g d.w.)	0.196 to 0.104 ↓ 0.196 to 0.100 ↓ 0.196 to 0.089 ↓	[115]
		- Extruded at 129 °C - Extruded at 135 °C - Extruded at 142 °C	Riboflavin (mg/100 g d.w.)	0.102 to 0.096 ↓ 0.102 to 0.087 ↓ 0.102 to 0.089 ↓	
Corn with pea and rosehip	- single-screw laboratory extruder, dosing speed of 18 rpm, feed rate of 3.51 kg/h, screw speed of 150 rpm, temperatures of barrel sections of 25/70/170/175 °C, die diameter of 3 mm.	- control (corn flour) - 10% rosehip - 10% rosehip + pea protein	Ascorbic Acid (mg/100 g d.w.)	5.57 to 4.50 ↓ 24.23 to 12.50 ↓ 30.63 to 7.40 ↓	[85]
		- control (corn flour) - 10% rosehip - 10% rosehip + pea protein	Vitamin C (mg/100 g d.w.)	20.47 to 16.81 ↓ 45.37 to 35.67 ↓ 56.81 to 33.62 ↓	
Soybean flour and acha (*Digitaria exilis*)	- single-screw laboratory extruder, barrel diameter of 40 mm, 3:1 compression ratio, dosing speed of 80 rpm, feed rate of 3.51 kg/h, feed moisture content 25%; screw speed: 150 rpm; barrel temperature: 150 °C, die diameter of 2 mm	- acha + soybean mix	Vitamin B2 (mg/100 g)	0.70 to 0.01 ↓	[116]
			Vitamin B6 (mg/100 g)	4.10 to 2.20 ↓	
			Vitamin C (mg/100 g)	0.01 to 0.01 =	
			Calcium (mg/100 g)	47.83 to 34.30 ↓	
			Potassium (mg/100 g )	360.00 to 176.00 ↓	
			Sodium (mg/100 g)	22.00 to 36.00 ↑	
			Iron (mg/100 g)	1.10 to 3.60 ↑	
			Zinc (mg/100 g)	1.40 to 0.09 ↓	
			Magnesium (mg/100 g)	109.13 to 81.40 ↓	
			Selenium (mg/100 g)	1.50 to 1.60 ↑	
Corn grits, turmeric, ginger, bay leaf, or laurel	- 13% moisture, temperature of 180 °C, screw speed of 700 rpm, 3 mm diameter die, feeding rate 3 kg/min.	- control (corn) - 3% laurel - 3% turmeric - 3% ginger - 3% mixture (1:1:1)	Vitamin B6 (mg/100 g)	0.04 to 0.03 ↓ 0.09 to 0.02 ↓ 0.09 to 0.08 ↓ 0.04 to 0.01 ↓ 0.06 to 0.05 ↓	[75]
		- control (corn) - 3% laurel - 3% turmeric - 3% ginger - 3% mixture (1:1:1)	Vitamin A (IU/100 g)	500.00 to 310.00 ↓ 669.74 to 316.00 ↓ 3448.6 to 445.00 ↓ 485.87 to 283.00 ↓ 1554.07 to 335.00 ↓	
		- control (corn) - 3% laurel - 3% turmeric - 3% ginger - 3% mixture (1:1:1)	Zinc (g/100 g)	0.74 to 0.49 ↓ 0.95 to 1.02 ↑ 0.99 to 1.08 ↑ 0.83 to 1.18 ↑ 0.99 to 1.29 ↑	
		- control (corn) - 3% laurel - 3% turmeric - 3% ginger - 3% mixture (1:1:1)	Potassium (g/100 g)	50.50 to 53.60 ↑ 60.30 to 55.80 ↓ 154.90 to 167.90 ↑ 55.70 to 70.30 ↑ 271.00 to 320.00 ↑	
		- control (corn) - 3% laurel - 3% turmeric - 3% ginger - 3% mixture (1:1:1)	Magnesium (g/100 g)	33.60 to 35.40 ↑ 34.80 to 37.70 ↑ 42.90 to 39.20 ↓ 37.60 to 38.90 ↑ 40.60 to 45.60 ↑	
		- control (corn) - 3% laurel - 3% turmeric - 3% ginger - 3% mixture (1:1:1)	Calcium (g/100 g)	40.10 to 33.90 ↓ 50.50 to 66.80 ↑ 56.20 to 76.70 ↑ 52.80 to 80.30 ↑ 66.70 to 93.70 ↑	
		- control (corn) - 3% laurel - 3% turmeric - 3% ginger - 3% mixture (1:1:1)	Iron (g/100 g)	3.15 to 4.88 ↑ 3.99 to 7.23 ↑ 14.84 to 26.01 ↑ 4.30 to 12.56 ↑ 5.73 to 29.56 ↑	
		- control (corn) - 3% laurel - 3% turmeric - 3% ginger - 3% mixture (1:1:1)	Sodium (g/100 g)	20.30 to 22.50 ↑ 17.50 to 20.90 ↑ 19.40 to 20.40 ↑ 18.50 to 21.60 ↑ 18.90 to 20.50 ↑	
		- control (corn) - 3% laurel - 3% turmeric - 3% ginger - 3% mixture (1:1:1)	Copper (g/100 g)	0.08 to 0.12 ↑ 0.04 to 0.13 ↑ 0.03 to 0.11 ↑ 0.07 to 0.15 ↑ 0.06 to 0.19 ↑	
Red sorghum	- twin-screw, co-rotating extruder	- decorticated sorghum flour	Calcium (mg/100 g d.w.)	17.00 to 10.00 ↓	[112]
			Iron (mg/100 g d.w.)	1.70 to 3.00 ↓	
			Zinc (mg/100 g d.w.)	0.60 to 1.00↓	
Rice, seeded banana, carambola pomace	- single-screw extruder, screw speed of 350 rpm, barrel temperature of 120 °C, feed moisture of 12 g/100 g	- optimal sample: low amylose rice: seeded banana: carambola pomace ratio: 65:25:10	Mg (mg/100 g)	10.62 to 14.54 ↑	[117]
			K (mg/100 g)	17.41 to 29.24 ↑	
Waxy rice, toasted defatted soy flour, spirulina, distilled monoglyceride, vitamin/ mineral premix	- twin-screw extruder, screw speed of 120 rpm, barrel temperature: 80, 80, 80, 25, 25 °C, feed moisture of 14%	- control - rice-soy + 2% spirulina - rice-soy + 4% spirulina - rice-soy + 6% spirulina - rice-soy + 8% spirulina	Vitamin A (IU/100 g)	9116.54 to 4167.32 ↓ 9184.63 to 4265.22 ↓ 9235.66 to 4406.64 ↓ 9325.62 to 4586.28 ↓ 9465.38 to 4735.22 ↓	[118]
		- control - rice-soy + 2% spirulina - rice-soy + 4% spirulina - rice-soy + 6% spirulina - rice-soy + 8% spirulina	Vitamin C (mg/100 g)	125.87 to 66.34 ↓ 126.57 to 65.94 ↓ 127.62 to 67.89 ↓ 131.86 to 64.38 ↓ 129.37 to 61.34 ↓	

= unchanged, ↑ increase, ↓ decrease.

### 3.3. Effects of Extrusion on Fibers

Different studies highlighted the effects of extrusion cooking on the total (TDF), soluble (SDF), and insoluble dietary fiber (IDF) content of extrudate products (Table 4). Arribas et al. [119] showed that extrusion determines a reduction in TDF of gluten-free extrudates snacks based on pea and rice flour. The authors reported different IDF and SDF after extrusion, depending on the addition level of the pea, as follows: at 20% pea, the IDF fraction was higher after the extrusion, while at 40%, SDF was predominant [119]. TDF registered higher values after the extrusion of lentil-based mixes with fiber-rich flours at 160 °C and 17% moisture, which led to a considerable rise in the SDF [120]. The temperature gradient, humidity, and shear applied in extrusion have a significant impact on the dietary fiber fractions composition [53]. Extrusion determined the rise of SDF proportion in a variety of vegetal by-products, leading to the diminishing of the IDF content and a decrease of TDF up to 6% [53]. García-Amezquita et al. [53] used different extrusion parameters for orange peels and stated that the reduction in IDF was correlated to the raise of SDF, generating thus enhanced functional properties. This can be due to the ability of extrusion to partially solubilize the IDF without total damage to the polymeric structure. Huang and Ma [121] investigated the impact of extrusion parameters of orange pomace and observed that the SDF raised up to 74% for the sample treated at 125 °C, 14% moisture, and 290 rpm compared to the raw one. Jan et al. [122] reported a significant negative effect of the increase in temperature, moisture, and screw speed on TDF content as a result of partial leaching of some fibers, especially hemicellulose. These results can be explained due to the formation of low molecular weight soluble fibers that are not recovered through alcoholic precipitation during TDF analysis [122].

Rashid et al. [123] reported that the increase in barrel temperature and screw speed resulted in an increase in the TDF content of wheat bran extrudates, mostly due to the rise of IDF. This can be explained by the higher level of soluble dietary fiber (SDF) content and the formation of resistant starch in thermal processing. The shear intensity during extrusion determines the depolymerization of starch and raises the linearity of amylopectin/amylose chains, contributing thus to the formation of resistant starch type III, which acts as fiber [124]. Another explanation is the formation of new covalent bonds with other macronutrients that resulted in insoluble compounds [125]. Extrusion generally enhances the proportion of SDF. Jing and Chi [126] showed an increase of 10.60% in SDF content in extruded soybean residues compared to the control. The SDF amount of the lupin seed coat raised up to 3-fold after extrusion, while IDF was considerably reduced [127].

**Table 4:** Effects of extrusion on the fiber content.

Food Matrix	Experiment Conditions		Effect			Ref.
	Extrusion Parameters	Sample	SDF (%)	IDF (%)	TDF (%)	
Lentil (*Lens culinaris* L.), wheat bran, apple fiber, NUTRIOSE^®^ corn fiber	- twin–screw extruder, screw speed of 500 rpm, medium feed rate of 50 kg/h, extrusion temperature of 160 °C, screw diameter of 32 mm.	- control - lentil + wheat bran + apple fiber - lentil + wheat bran + NUTRIOSE^®^ - lentil + apple fiber + NUTRIOSE^®^ - lentil + apple fiber + corn fiber	0.11 to 0.55 ↑ 0.12 to 0.75 ↑ 0.27 to 0.31 ↑ 0.23 to 0.25 ↑ 0.52 to 0.63 ↑	12.31 to 7.65 ↓ 9.20 to 8.64 ↓ 11.12 to 7.09 ↓ 8.70 to 7.50 ↓ 10.81 to 9.97 ↓	12.42 to 8.20 ↓ 9.32 to 9.39 ↑ 11.39 to 7.49 ↓ 10.13 to 7.75 ↓ 10.15 to 10.23 ↑	[120]
Soybean by-product	- twin-screw extruder, extrusion temperature 114.57 °C, moisture of 31.37%, screw speed of 182.95 rpm	- soybean by-product	2.05 to 12.65 ↑	60.82 to 50.39 ↓	63.03 to 63.07 ↓	[126]
Rice, pea, carob	- twin–screw extruder, medium rate of 25 kg/h, screw diameter of 25 mm, final barrel temperature of 125 °C, speed of 900–950 rpm; - water at a rate of 2.50 (sample without carob), 3.00 (sample with 5% carob), and 3.22 kg/h (sample with 10% carob).	- 20% pea 0% carob - 20% pea 5% carob - 20% pea 10% carob - 40% pea 0% carob - 40% pea 5% carob - 40% pea 10% carob	2.58 to 1.06 ↓ 3.77 to 3.21 ↓ 5.68 to 2.91 ↓ 4.07 to 3.59 ↓ 3.66 to 3.78 ↑ 3.99 to 4.72 ↑	2.38 to 1.87 ↓ 4.08 to 2.41 ↓ 5.03 to 4.76 ↓ 6.87 to 3.81 ↓ 10.48 to 5.60 ↓ 11.05 to 5.58 ↓	4.96 to 2.93 ↓ 7.84 to 5.61 ↓ 10.71 to 7.67 ↓ 10.94 to 7.40 ↓ 14.14 to 9.39 ↓ 15.03 to 10.31 ↓	[119]
Chickpea–rice, passion fruit, and Fibersol^®^	- twin-screw laboratory extruder, at a feed rate of 20 kg/h, screw diameter of 12 mm, last barrel section temperature of 140 °C, die diameter of 3.5 mm, screw speed of 500 rpm, 17% moisture.	- control - 20% passion fruit, 5% Fibersol^®^ - 12.5% passion fruit, 5% Fibersol^®^ - 5% passion fruit, 5% Fibersol^®^ - 20% passion fruit, 7.5% Fibersol^®^ - 12.5% passion fruit, 7.5% Fibersol^®^ - 5% passion fruit, 7.5% Fibersol^®^ - 20% passion fruit, 10% Fibersol^®^ - 12.5% passion fruit, 10% Fibersol^®^ - 5% passion fruit, 10% Fibersol^®^	1.57 to 4.85 ↑ 2.82 to 4.12 ↑ 2.47 to 5.25 ↑ 2.78 to 6.50 ↑ 1.56 to 3.03 ↑ 4.81 to 6.54 ↑ 3.66 to 4.77 ↑ 5.23 to 4.21 ↑ 5.82 to 4.84 ↓ 4.19 to 4.65 ↑	8.50 to 8.13 ↓ 11.74 to 14.16 ↑ 10.18 to 7.62 ↓ 7.45 to 10.23 ↑ 8.57 to 5.69 ↓ 6.08 to 9.58 ↑ 5.32 to 6.10 ↑ 8.63 to 8.98 ↑ 10.92 to 9.36 ↓ 4.46 to 9.04 ↑	10.07 to 12.98 ↑ 14.56 to 18.29 ↑ 12.65 to 12.87 ↑ 10.22 to 16.73 ↑ 10.13 to 8.72 ↓ 10.88 to 16.13 ↑ 9.10 to 10.88 ↑ 13.86 to 12.85 ↓ 16.74 to 14.20 ↓ 8.65 to 13.69 ↑	[107]
Buckwheat	- twin-screw extruder. screw diameter of 20 mm, barrel temperature: 100–160 °C, moisture content of 48%.	- buckwheat at 100 °C - buckwheat at 120 °C - buckwheat at 140 °C - buckwheat at 160 °C	3.13 to 3.33 ↑ 3.13 to 3.48 ↑ 3.13 to 3.87 ↑ 3.13 to 4.14 ↑	7.06 to 6.64 ↓ 7.06 to 6.47 ↓ 7.06 to 6.15 ↓ 7.06 to 5.77 ↓	10.18 to 9.93 ↓ 10.18 to 9.95 ↓ 10.18 to 10.02 ↓ 10.18 to 9.91 ↓	[128]
Non-wheat flours	- barrel temperature: feeding zone 50 °C, compression zone 120 °C and cooking zone 220 °C, moisture content of 27%.	- Barley - Rye - Triticale - Oat - Sorghum - Millet	-	9.59 to 8.95 ↓ 9.70 to 8.82 ↓ 13.51 to 13.95 ↑ 8.30 to 7.18 ↑ 10.04 to 9.41 ↓ 1.96 to 2.57 ↑	16.80 to 17.08 ↑ 14.39 to 13.03 ↓ 19.93 to 18.37 ↓ 12.42 to 12.75 ↑ 13.52 to 13.24 ↓ 5.12 to 4.78 ↓	[129]
Lupin seed coat	- co-rotating intermeshing twin-screw extruder, feed rate of 4 kg/h, variable barrel temperatures (120–150 °C), screw speed of 400 rpm, moisture of 40%.	- lupin seed coat at 120 °C - lupin seed coat at 135 °C - lupin seed coat at 150 °C	4.42 to 7.04 ↑ 4.42 to 6.73 ↑ 4.42 to 7.55 ↑	91.00 to 87.41 ↓ 91.00 to 87.25 ↓ 91.00 to 85.49 ↓	95.42 to 94.45 ↓ 95.42 to 93.98 ↓ 95.42 to 93.04 ↓	[90]
Soybean, canola, sunflower cakes	- twin-screw extruder, temperature profile of 40/60/80/100/130 °C moisture of 17%, feeding rate of 13.2 Kg/h, screw speed of 500 rpm.	- soybean cake - canola cake - sunflower cake	7.6 to 5.3 ↓ 11.1 to 14.3 ↑ 6.8 to 11.1 ↑	14.6 to 14.4 ↓ 24.6 to 24.7 ↑ 34.3 to 35.6 ↑	22.5 to 19.7 ↓ 35.8 to 39.1 ↑ 41.1 to 46.7 ↑	[91]
Nut shell	- twin-screw co-rotating extruder, feed rate of 7.79 kg/h dry matter, screw speed of 150 rpm, barrel temperature of 70 °C.	- optimal sample	0.00 to 3.07	75.41 to 76.03	75.41 to 79.1	[92]
Oat bran	- twin–screw extruder, speed of 150 rpm, feed rate of 18 kg/h, barrel temperature of 100/120/140/160 °C, 10–30% moisture.	- oat bran 10% moisture, 100 °C - oat bran 20% moisture, 100 °C - oat bran 30% moisture, 100 °C	8.90 to 9.90 ↑ 8.90 to 9.50 ↑ 8.90 to 9.50 ↑	-	-	[130]
		- oat bran 10% moisture, 160 °C - oat bran 20% moisture, 160 °C - oat bran 30% moisture, 160 °C	8.90 to 12.40 ↑ 8.90 to 11.10 ↑ 8.90 to 11.00 ↑			
Rice, milk powder, potato starch, corn starch, soya, cranberry, carrot, beetroot, teff	- twin–screw extruder, screw speed of 275–350 rpm, feed rate of 15–25 kg/h, barrel temperature of 80–150 °C, 12% moisture.	- rice - rice + cranberry - rice + carrot - rice + beetroot - rice + teff	-	-	16.76 to 112.60 ↑ 7.30 to 33.20 ↑ 10.02 to 28.32 ↑ 2.93 to 23.27 ↑ 139.20 to 190.80 ↑	[131]
Chokeberry pomace with corn	- single-screw extruder, 14% moisture content in the mixture before extrusion, screw speed of 190 rpm, compression ratio of 1:3, temperature profile of 100/120/140 °C, die diameter of 4 mm.	- chokeberry pomace + corn	-	-	42.00 to 37.83 ↓	[132]

SDF—soluble dietary fiber, IDF—insoluble dietary fiber, TDF—total dietary fiber, ↑ increase, ↓ decrease.

The screw speed and moisture of the sample temperature profile used in extrusion were proved to be the main factors influencing seed coat fiber composition, as follows: higher temperature, pressure, screw speed, and moisture content seem to result in higher SDF content, while after a certain parameter point, SDF content seems to be stagnant or even decrease [127]. This may be a result of glycosidic linkage depolymerization in polysaccharides and changes in its solubility and structure, thus modifying the functional properties of the extruded fiber [127]. Extrusion processing led to the rise of SDF fraction of some fiber-rich ingredients like pea seed coat, sugar beet pulps, soybean by-product [126], carrot pomace [133], orange be-product [121], wheat bran [123], rice and rye bran [134]. The greatest SDF to TDF ratio of the wheat or rye-bran-containing snacks was reported at high screw speed (400 rpm), raised temperature (130 °C), and low sample moisture (24% for wheat and 30% for rye) [134].

### 3.4. Effects of Extrusion on the Biological Activity

The oxidative stress caused by reactive oxygen species (ROS) and reactive nitrogen species (RNS) is responsible for many disorders of the biological systems by affecting DNA and promoting lipid and protein oxidation. The intake of antioxidants from food could limit the negative effects of ROS and RNS by inhibiting their action [135]. The main compounds from plants that have antioxidant properties are polyphenols like phenolic acids, flavonoids, anthocyanins, lignans and stilbenes, terpenes and terpenoids, vitamins like E and C [135], and some minerals such as zinc, copper, and selenium [136,137,138]. Some of these compounds, especially polyphenols, and carotenoids (tetraterpenoids), also have “anti-inflammatory, antibacterial, antiviral, anti-aging, and anticarcinogenic properties” [135]. Special attention is given to the antioxidant fibers, which are polysaccharides with bound polyphenols that present significant antioxidant activity [139].

Many studies investigated the effects of extrusion on the antioxidant activity of cereals, fruits, legumes, vegetables, and seeds (Table 5). Allai et al. [140] found that the extrusion of wheat, barley, Indian horse chestnut flour, and their mixes caused the reduction in antioxidant activity compared to the raw materials due to the changes in the bioactive compounds’ chemical structure and polymerization reactions. Another study reported a positive influence of the screw speed on the chicory–rice blend’s antioxidant activity, while the increase in moisture led to lower antioxidant properties of the mix [141]. The radical scavenging activity of blackcurrant pomace decreased by 11% after extrusion, the main factors influencing this result being the screw spend and barrel temperature [142]. On the other hand, Ortak et al. [88] stated that the temperature and screw speed did not significantly influence the antioxidant activity of corn–carrot snacks but obtained a decrease compared to the unprocessed mix. These differences among the results presented in the literature are mainly due to the different processing conditions and the food matrix composition. It is known that high shear stress is responsible for phenolics structure breaking, while a temperature greater than 80 °C promotes structure changes of the thermolabile phenolics and polymerization [88].

Witczak et al. [143] also obtained a reduction in the antioxidant compounds level in corn–cherry and corn–blackcurrant pomace mixes as a result of anthocyanins damage caused by heat, presence of enzymes, and processing pressure applied. The decrease in phenolics content of corn–lucerne mixes after extrusion was correlated to the lowering of the antioxidant activity, according to the data presented by Igual et al. [95].

**Table 5:** Effects of extrusion on the antioxidant activity.

Food Matrix	Experiment Conditions		Effect on the Antioxidant Activity	Ref.
	Extrusion Parameters	Sample		
Rice, pea, and carob fruit	- twin–screw extruder, medium rate of 25 kg/h, screw diameter of 25 mm, final barrel temperature of 125 °C, speed of 900–950 rpm; - water at a rate of 2.50 (sample without carob), 3.00 (sample with 5% carob), and 3.22 kg/h (sample with 10% carob).	- 20% pea 0% carob - 20% pea 5% carob - 20% pea 10% carob - 40% pea 0% carob - 40% pea 5% carob - 40% pea 10% carob	8.35 to 9.81 ^1^ ↑ 9.32 to 10.97 ^1^ ↑ 10.20 to 12.00 ^1^ ↑ 10.45 to 9.69 ^1^ ↓ 11.46 to 10.95 ^1^ ↓ 11.83 to 11.63 ^1^ ↓	[22]
Cassava-soy composite with grape pomace	- co-rotating twin-screw extruder, 5 heating areas at 60/80/100/140/140 °C, water rate of 3 L/h, feed rate of 25 kg/h, die diameter of 3 mm, screw speed of 200 rpm.	- 0% grape pomace - 10% grape pomace - 20% grape pomace	7.7 to 5.5 ^2^ ↓ 10.6 to 8.5 ^2^ ↓ 13.5 to 9.8 ^2^ ↓	[74]
Corn, carrot powder, ascorbic acid	- single-screw extruder, temperature profiles of 135/170/170 °C, 4:1 compression ratio screw, screw speed of 100 rpm, feed rate of 15 rpm.	- control - 4% carrot powder - 6% carrot powder - 8% carrot powder	1.21 to 0.88 ^2^ ↓ 2.06 to 1.76 ^2^ ↓ 2.04 to 1.73 ^2^ ↓ 2.11 to 2.32 ^2^ ↓	[84]
Maize and bean	- single-screw extruder, 19 mm diameter screw, 3 mm die diameter, barrel temperature of 164 °C, screw speed of 187 rpm.	- 70% maize + 30% bean flour	0.09 to 0.12 ^3^ ↑ 0.19 to 0.33 ^4^ ↑	[72]
Quinoa flour	- moisture adjusted at 18%; - temperature of 75 °C, 105 °C, and 135 °C, screw rotation speed of 251–253 rpm, three nozzles of 2.6 mm diameter.	- quinoa flour	13.16 to 19.72 ^2^ ↑ 3.87 to 5.32 ^5^ ↑	[67]
Rice	- co-rotating twin-screw extruder, 15.5% and 16% of feed moisture, 159 and 150 °C for the last barrel zone temperature for black and red rice, respectively	- black rice - red rice	1.69 to 0.68 ^1^ ↓ 0.66 to 0.24 ^1^ ↓	[66]
		- black rice - red rice	0.28 to 0.09 ^5^ ↓ 0.06 to 0.03 ^5^ ↓	
Potato	- single-screw extruder, die diameter of 0.5 mm, feeding speed of 38 rpm, screw speed of 120 rpm, temperature profile of 60/70/80 °C), dough moisture of 40–45%; - the extruded dough was fried in rapeseed oil for 15–20 s at 180 °C	- Control - Salad Blue - Blue Congo - Valfi - Herbie 26 - Control - Salad Blue - Blue Congo - Valfi - Herbie 26	1.08 to 0.67 ^2^ ↓ 0.57 to 1.16 ^2^ ↑ 2.18 to 1.34 ^2^ ↓ 1.85 to 1.16 ^2^ ↓ 0.52 to 1.17 ^2^ ↑ 0.61 to 0.22 ^5^ ↓ 0.26 to 0.60 ^5^ ↑ 1.12 to 0.68 ^5^ ↓ 0.83 to 0.59 ^5^ ↓ 0.27 to 0.46 ^5^ ↑	[144]
Corn grits, turmeric, ginger, bay leaf, or laurel	- 13% moisture, temperature of 180 °C, screw speed of 700 rpm, 3 mm diameter die, feeding rate 3 kg/min.	- control (corn) - 3% laurel - 3% turmeric - 3% ginger - 3% mixture (1:1:1)	12.89 to 15.59 ^6^ ↑ 76.01 to 64.44 ^6^ ↓ 69.54 to 50.09 ^6^ ↓ 47.36 to 30.98 ^6^ ↓ 61.97 to 37.02 ^6^ ↓	[75]
Mustard meal concentrate, wheat flour	- moisture levels of 12–18%, twin-screw extruder, barrel temperatures of 100–150 °C, screw speed 250–350 rpm.	- 5% mustard meal - 10% mustard meal - 15% mustard meal	48.41 to 51.06 ^6^ ↑ 62.11 to 64.97 ^6^ ↑ 71.09 to 73.36 ^6^ ↑	[81]
Corn flour with pea protein, broccoli, lucerne, beetroot, rosehip, turmeric, chili, paprika, and basil	- single-screw laboratory extruder, screw diameter of 19 mm, 4 heating areas 50/100/140/140 °C, die diameter of 4 mm, screw speed of 100 rpm, and feeding speed of 20 rpm;	- Control (corn) - 2% pea - 5% broccoli - 5% lucerne - 15% beetroot - 15% rosehip - 2% chili - 2% turmeric - 2% paprika - 2% basil	90.33 to 53.26 ^7^ ↓ 91.16 to 59.44 ^7^ ↓ 109.77 to 83.42 ^7^ ↓ 105.67 to 83.48 ^7^ ↓ 254.39 to 250.48 ^7^ ↓ 282.59 to 329.20 ^7^ ↑ 97.97 to 63.68 ^7^ ↓ 101.59 to 64.00 ^7^ ↓ 97.25 to 70.02 ^7^ ↓ 142.67 to 104.15 ^7^ ↓	[82]
Corn and lucerne	- single-screw laboratory extruder, barrel diameter of 19 mm, 3:1 compression ratio, dosing speed of 18 rpm, feed rate of 3.4 kg/h, rotation speed of 150 rpm, temperatures of barrel sections of 25/70/170/175 °C, nozzle diameter of 3 mm.	- 2.5% lucerne - 5% lucerne - 7.5% lucerne - 10% lucerne - 12.5% lucerne - 15% lucerne	169.00 to 161.00 ^7^ ↓ 196.00 to 180.00 ^7^ ↓ 228.00 to 198.00 ^7^ ↓ 262.00 to 216.00 ^7^ ↓ 320.00 to 241.00 ^7^ ↓ 358.00 to 257.00 ^7^ ↓	[95]
Corn with pea and rosehip	- single-screw laboratory extruder, barrel diameter of 19 mm, 3:1 compression ratio, dosing speed of 18 rpm, feed rate of 3.51 kg/h, rotation speed of 150 rpm, temperatures of barrel sections of 25/70/170/175 °C, nozzle diameter of 3 mm.	- control (corn flour) - 10% rosehip - 10% rosehip + pea protein	1.73 to 0.00 ^7^ ↓ 201.00 to 14.01 ^7^ ↓ 64.70 to 12.30 ^7^ ↓	[85]
Corn, brewer’s spent grain, sugar beet pulp, apple pomace	- blends with 15% moisture content - single-screw extruder, temperature steps of 135/170/170 °C, compression ratio of 4:1, die of 4 mm diameter.	- Corn grits - 5% brewer’s spent grain - 10% brewer’s spent grain - 15% brewer’s spent grain - 5% sugar beet pulp - 10% sugar beet pulp - 15% sugar beet pulp - 5% apple pomace - 10% apple pomace - 15% apple pomace	17.78 to 19.51 ^6^ ↑ 16.71 to 18.85 ^6^ ↑ 16.08 to 17.36 ^6^ ↑ 15.41 to 16.97 ^6^ ↑ 16.51 to 18.51 ^6^ ↑ 15.07 to 17.58 ^6^ ↑ 13.33 to 16.64 ^6^ ↑ 24.65 to 36.67 ^6^ ↑ 31.06 to 54.80 ^6^ ↑ 38.31 to 78.11 ^6^ ↑	[83]
Pearl millet with almond cake	- twin-screw extruder, with circular die of 3 mm diameter, temperature of 60 °C in the first step and 80 °C in the second one, 120 °C for the last step, 450 rpm speed	- 80% pearl millet + 20% almond cake	89.93 to 89.74 ^6^ ↓	[76]
Corn grits, germinated and dehulled chickpea, tomato powder, skim milk	- single-screw extruder, temperatures steps at 100/160/180 °C, feeding screw speed 160 rpm, screw speed 250 rpm, screw compression 4:1, die diameter of 3 mm	- control - 10% chickpea - 20% chickpea - 30% chickpea	41.54 to 45.46 ^6^ ↑ 50.81 to 53.82 ^6^ ↑ 53.32 to 54.94 ^6^ ↑ 55.26 to 56.33 ^6^ ↑	[86]
Corn grits with cocoa husk	- single-screw extruder, 4:1 screw, die diameter of 4 mm, temperature profile: 135/170/170 °C	- control - 5% cocoa husk - 10% cocoa husk - 15% cocoa husk	11.03 to 11.25 ^6^ ↑ 15.24 to 20.50 ^6^ ↑ 19.60 to 25.76 ^6^ ↑ 23.47 to 33.08 ^6^ ↑	[87]
Corn grits with carrot pulp	- twin-screw extruder, die diameter of 3 mm, feeding rate of 36 ± 1 g/min, temperature profile 1 (80/90/100/130/120 °C) and 2 (80/105/130/160/130 °C), screw speed of 125 or 225 rpm	- temperature profile 1, 125 rpm - temperature profile 1, 225 rpm - temperature profile 2, 125 rpm - temperature profile 1, 225 rpm	6.80 to 5.34 ^5^ ↓ 6.80 to 4.91 ^5^ ↓ 6.80 to 5.20 ^5^ ↓ 6.80 to 5.09 ^5^ ↓	[88]
Corn grits with tomato powder	- single-screw polytrophic extruder, screw compression ratio of 3:1, barrel temperatures of 125–145–135°C, die diameter of 3 mm, screw speed of 120 rpm.	- Control - 5% tomato - 10% tomato - 15% tomato - 20% tomato - 25% tomato - 30% tomato	0.2 to 0.3 ^5^ ↑ 0.3 to 1.3 ^5^ ↑ 0.9 to 3.6 ^5^ ↑ 1.1 to 3.7 ^5^ ↑ 1.2 to 3.9 ^5^ ↑ 2.0 to 4.2 ^5^ ↑ 2.0 to 4.8 ^5^ ↑	[89]

^1^—measured by ORAC (oxygen radical absorbance capacity) method and the results expressed as μmol TE/g d.w.; ^2^—measured by ABTS method and the results expressed as μmol TE/g; ^3^—measured by ABTS (2,2′-Azino-bis(3-ethylbenzothiazoline-6-sulfonic acid) diammonium salt radical cation) and expressed as mg/mL; ^4^—measured by DPPH (2,2-diphenyl-1-picrylhydrazyl) and expressed as mg/mL; ^5^—measured by DPPH and expressed as μmol TE/g d.w.; ^6^—measured by DPPH method and expressed as %, ^7^—measured by DPPH and expressed as mg TE/100 g d.w., ↑ increase, ↓ decrease.

The effects of extrusion on the corn snacks enriched with pea protein, broccoli, lucerne, beetroot, rosehip, turmeric, chili, paprika, or basil antioxidant activity revealed a decrease by 2 to 41% compared to the raw mixes, except for the rosehip-containing sample which exhibited an opposite trend [82]. The authors attributed these results to the thermal damage of phenolics, to the better extraction of polyphenols due to the low-molecular phenolics formation through the disintegration of complex molecules, and/or to the Maillard reactions between sugars and amino acids, which led to the formation of new compounds with antioxidant properties [82].

Contrary to other results, Chakraborty et al. [81] observed an increase in the radical scavenging activity of wheat–mustard meal mixes after extrusion. Arribas et al. [22] reported increases or decreases in the antioxidant activity of rice–carob–pea mixes after extrusion, depending on the amount of pea used; at 20%, higher values compared to the raw blends were obtained, while at 40%, the antioxidant activity decreased. Thus, the impact of extrusion on the antioxidant activity of snacks depends on the chemical composition of the food matrix, mainly the presence of proteins, carbohydrates, and aromatic compounds apart from phenolics [22]. Free phenolics’ antioxidant activity was significantly affected by the extrusion of maize–bean mixes, while the antioxidant activity of bound phenolics was not affected and was higher compared to the free phenolics one [72]. These findings could be related to the greater amount of highly reactive antioxidant molecules found in bound phenolics compared to the free ones [72]. The extrusion of quinoa flour determined the increase in its antioxidant activity mainly due to the formation of Maillard products and the leaching of conjugated phenolics under high-pressure conditions [67]. The cultivating conditions and the genetic factors affect the phenolic profile of the seeds [67], which will further influence the antioxidant properties of the final product.

The metabolization of carbohydrates depends on the enzymatic activity, especially α-amylase and α-glucosidase; their inhibition led to the attenuation of pre-diabetic and diabetic disease [71]. Yao and Ren [145] observed that the extrusion processing of adzuki beans resulted in a significant improvement in antidiabetic activities (an increase of more than 300% compared to the raw material). The authors demonstrated that along with anthocyanins and other phenolics, proteins also exhibited an inhibition effect on α-glucosidases [145]. The extrusion of sesame seeds by-product determined the increase in α-amylase and α-glucosidase enzymes inhibition effects of the beverage prepared from it, with a more pronounced trend on α-glucosidase due to the affinity of phenolics from sesame toward this enzyme [146]. Hemp seeds presented a greater inhibitory effect on α-glucosidase enzyme after extrusion, a trend that was correlated with the phenolics content and antioxidant activity [71]. Furthermore, the authors also reported that extrusion improved the inhibition of Acetylcholinesterase which was proven to promote Alzheimer’s disease [71]. Qiao et al. [147] investigated the impact of extrusion on sweet potato soluble dietary fiber and revealed that the glucose, cholesterol, and bile salt absorption increased compared to the untreated sample, while the α-amylase and pancreatic lipase activity were strongly inhibited by the extruded sample due to the modification of the porous fiber matrix, which determined raised surface area that can absorb higher quantities of enzymes, glucose, and oil.

The contradictory data reported in the literature on the effects of extrusion on antioxidant activity are mainly due to the differences in food matrix chemical composition and extrusion process conditions, such as screw speed, pressure, and barrel temperature, but also to the interactions that occur between components during processing. The anti-diabetic properties were proved to be enhanced by the extrusion of vegetal food matrices.

## 4. Conclusions and Further Perspectives

Extrusion is a convenient processing technique to create novel snacks enriched with functional ingredients from vegetal sources. The most common food matrices used to obtain functional snacks include cereals, pseudo-cereals, fruits and vegetables, legumes, seeds, or their by-products. The processing conditions, namely screw speed, temperature profile, and pressure, are the main factors influencing the bioactive profile of the final products. Furthermore, the chemical composition and structure of the food matrix dictate the magnitude of changes in the phenolics, terpenes, vitamins, minerals, and fiber content of the snacks. Increases or decreases of the phenolics, carotenoids, minerals, and fibers were reported in the literature, the discrepancies being attributed to the differences in the processing equipment and operating parameters, chemical composition of the ingredients, and/or the analytical methods used for qualification of the bioactive compounds. Generally, the vitamin and thermolabile phenolics content decreased after extrusion, temperature being the main factor responsible for that.

There are few papers regarding the content of terpenes of the food matrices before and after extrusion, most of them being focused on the carotenoid profile. Thus, there is a need for in-depth research on this topic, as well as on the interactions between the bioactive components during extrusion. Furthermore, some studies regarding the industrial application and the effects of extrusion at the industrial level would be recommended since the majority of the papers existing in the literature present laboratory-scale studies.

## Data Availability

Not applicable.
